# Changes in Overall Survival over Time for Patients with de novo Metastatic Breast Cancer

**DOI:** 10.3390/cancers13112650

**Published:** 2021-05-28

**Authors:** Toshiaki Iwase, Tushaar Vishal Shrimanker, Ruben Rodriguez-Bautista, Onur Sahin, Anjali James, Jimin Wu, Yu Shen, Naoto T. Ueno

**Affiliations:** 1 Section of Translational Breast Cancer Research, Department of Breast Medical Oncology, The University of Texas MD Anderson Cancer Center, 1515 Holcombe Boulevard, Houston, TX 77030, USA; tiwase@mdanderson.org (T.I.); tushaar.shrimanker@greenwichhospital.org (T.V.S.); rrodriguez15@mdanderson.org (R.R.-B.); osahin1@mdanderson.org (O.S.); ajames2@mdanderson.org (A.J.); 2 Morgan Welch Inflammatory Breast Cancer Research Program and Clinic, The University of Texas MD Anderson Cancer Center, 1515 Holcombe Boulevard, Houston, TX 77030, USA; 3 Department of Biostatistics, The University of Texas MD Anderson Cancer Center, 1515 Holcombe Boulevard, Houston, TX 77030, USA; jwu5@mdanderson.org (J.W.); yshen@mdanderson.org (Y.S.)

**Keywords:** breast neoplasms, neoplasm metastasis, inflammatory breast neoplasms, survival analysis

## Abstract

**Simple Summary:**

The present study aimed to clarify the change in survival for de novo metastatic breast cancer over time. We found that overall survival significantly improved over time for the estrogen-receptor-positive, HER2-positive subtype, and exhibited a tendency toward improvement over time for the estrogen-receptor-negative, HER2-positive subtype. These results underscored the contribution of HER2-targeted therapy to survival.

**Abstract:**

The purpose of this study was to determine the change in overall survival (OS) for patients with de novo metastatic breast cancer (dnMBC) over time. We conducted a retrospective cohort study with 1981 patients with dnMBC diagnosed between January 1995 and December 2017 at The University of Texas MD Anderson Cancer Center. OS was measured from the date of diagnosis of dnMBC. OS was compared between patients diagnosed during different time periods: 5-year periods and periods defined according to when key agents were approved for clinical use. The median OS was 3.4 years. The 5- and 10-year OS rates improved over time across both types of time periods. A subgroup analysis showed that OS improved significantly over time for the estrogen-receptor-positive/HER2-positive (ER+/HER2+) subtype and exhibited a tendency toward improvement over time for the ER-negative (ER−)/HER2+ subtype. In addition, median OS was significantly longer in patients with non-inflammatory breast cancer (*p* = 0.02) and patients with ER+ disease, progesterone-receptor-positive disease, HER2+ disease, lower nuclear grade, locoregional therapy, and metastasis to a single organ (all *p* < 0.0001). These findings showed that OS at 5 and 10 years after diagnosis in patients with dnMBC improved over time. The significant improvements in OS over time for the ER+/HER2+ subtype and the tendency toward improvement for the ER−/HER2+ subtype suggest the contribution of HER2-targeted therapy to survival.

## 1. Introduction

De novo metastatic breast cancer (dnMBC) is breast cancer that manifests metastasis at the time of initial diagnosis; dnMBC is different from recurrent metastatic breast cancer (rMBC). In the US, dnMBC accounted for approximately 6% of all breast cancer cases between 2005 and 2011 [1].

A previous retrospective study comparing clinical characteristics between patients with dnMBC and rMBC showed that patients with dnMBC tended to have higher rates of hormone receptor (HR)-negative disease, bone metastasis, liver metastasis, and a lower rate of central nervous system metastasis [2]. In survival analysis of 643 patients with dnMBC and 2881 with rMBC, the dnMBC patients had longer median overall survival (OS) (39.2 months vs. 27.2 months) [3]. Plausible reasons for the better OS of patients with dnMBC include that (1) dnMBC is chemonaïve, and a lower percentage of patients with dnMBC than with rMBC had originally chemo-resistant disease, and (2) general clinical characteristics of dnMBC patients such as bone-only metastasis and oligo-metastasis are dormant.

Although dnMBC differs clinically from rMBC, the general treatment strategy for dnMBC follows the rMBC treatment algorithm. In rMBC, the introduction of newly developed chemotherapy and targeted therapy has contributed to improvement in survival over time. For instance, the introduction of drugs targeting human epidermal growth factor receptor 2 (HER2) drastically improved the survival of patients with rMBC [4]. However, little is known about how advances in treatment have changed survival for patients with dnMBC. Moreover, the impact of biological features and locoregional interventions on survival in patients with dnMBC needs to be elucidated. Clarifying these unknowns will contribute to a better understanding of dnMBC’s biology and guide future research directions.

We hypothesized that the OS of patients with dnMBC has improved over time along with the development in drug therapy. The primary objective of this study was to test our hypothesis. The secondary objective was to identify biological and clinical prognostic factors for dnMBC.

## 2. Materials and Methods

### 2.1. Inclusion Criteria

**Figure 1:**
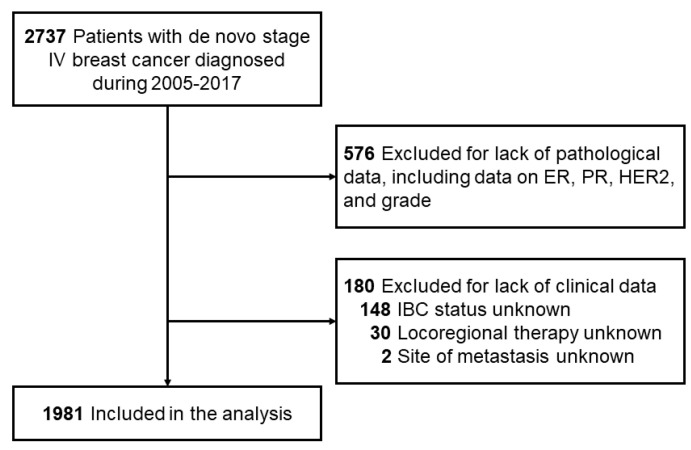
Patient selection process.

We conducted a retrospective cohort study with prospectively collected data designed to determine the change in OS over time for dnMBC. We defined dnMBC as breast cancer with histologically proven distant metastasis at the time of initial diagnosis. Using this definition of dnMBC, we retrospectively searched the Breast Cancer Electronic Medical Record Management System at The University of Texas MD Anderson Cancer Center for patients diagnosed with dnMBC from January 1995 through December 2017. This search identified 2737 potentially eligible patients with dnMBC (Figure 1). Of these patients, 576 patients were excluded because of insufficient pathological information, including data on estrogen receptor (ER) status, progesterone receptor (PR) status, HER2 status, and grade. An additional 180 patients were excluded because of lack of information about inflammatory breast cancer (IBC, N = 148), locoregional therapy (N = 30), or site of metastasis (N = 2). In total, 1981 patients with dnMBC were eligible for analysis. The primary outcome was OS, which was defined as the time from the date of the initial diagnosis until the date of death from any cause. The patient’s time to death was censored at the last follow-up if the patient was alive without an event occurrence at the last follow-up.

### 2.2. Definition of Time Periods

To determine the changes in OS over time, two types of time periods were used: (1) time periods based on the date of approval of key agents by the U.S. Food and Drug Administration (FDA) and (2) 5-year periods. The key agents were trastuzumab, approved in 1999; fulvestrant, approved in 2002; ado-trastuzumab emtansine, approved in 2013; pertuzumab, approved in 2013. For triple-negative breast cancer (TNBC), PARP inhibitors were potential key drugs; however, we excluded PARP inhibitors because their potential to contribute to OS improvement seemed limited given the low prevalence of *BRCA1* and *BRCA2* mutations in previous reports [5,6,7].

### 2.3. Definitions of Clinical and Pathological Variables

Cases of dnMBC were defined as positive for ER or PR if at least 1% of cells expressed the hormone receptor by immunohistochemistry (IHC). HER2 positivity was determined according to the American Society of Clinical Oncology/College of American Pathologists guidelines at the time of pathological evaluation. IBC was a clinical diagnosis made by a multidisciplinary team consisting of a medical oncologist, surgical oncologist, radiologist, and nurse; IBC was diagnosed when patients exhibited one or more of a set of specific clinical manifestations, including the history of rapid onset of breast erythema, edema and/or *peaud’orange*, and/or warm breast, with or without an underlying palpable mass. A history of flattening, crusting, or retraction of the nipple was also considered. Locoregional treatment was defined as surgical resection of the primary tumor by any method and radiation therapy delivered to the area of the breast and regional lymph nodes regardless of the dose and the frequency of the treatment. Metastasis to a single organ was defined as metastasis in only one organ, such as bone, lung, brain, or soft tissue, including distant lymph nodes (but not regional lymph nodes), confirmed by imaging and pathology. Patients with multiple sites of metastasis in the same organ were considered to have metastasis to a single organ if that was the only organ involved.

### 2.4. Statistical Considerations

Data were summarized using standard descriptive statistics, such as mean, standard deviation, median, and range for continuous variables and frequency, and proportion for categorical variables. OS duration was estimated using the Kaplan–Meier method, and OS was compared between or among groups using the log-rank test. Univariate and multivariate Cox regression models were applied to assess the effect of variables of interest on OS. The model was selected by the backward method. We considered two-sided *p* < 0.05 significant. All computations were carried out in SAS 9.4 (SAS Institute Inc., Cary, NC, USA). The study was approved by the Institutional Review Board of The University of Texas MD Anderson Cancer Center before data collection was initiated (protocol number: PA17-0613).

## 3. Results

### 3.1. Patient Characteristics

Table 1 shows the clinical characteristics of the patients included in the study. The median age was 52 years. Approximately 70% of the patients had ER-positive (ER+) disease, and 28% had HER2-positive (HER2+) disease. Fifteen percent of the patients had TNBC. Seventeen percent of the patients had IBC. Thirty-nine percent of the patients received locoregional therapy, and 62% had metastasis to a single organ.

### 3.2. OS by Disease and Treatment Characteristics

The median follow-up time was 7.4 years (95% confidence interval [CI]: 6.99–8.22). A total of 1365 of the 1981 patients died by the end of the study period. The median OS interval was 3.4 years (95% CI: 3.18–3.64) (Figure S1). The median OS interval was significantly longer in patients with non-IBC (vs. IBC) (*p* = 0.0210); median OS was also significantly longer in patients with ER+ disease (vs. ER−), PR+ disease (vs. PR negative [PR−]), HER2+ disease (vs. HER2−), lower nuclear grade, locoregional therapy (vs. none), and metastasis to a single organ (vs. multiple organs) (all *p* < 0.0001) (Figure S2A–D,F–H). The Kaplan–Meier curves crossed at around 7 years for IBC (Figure S2A), 10 years for ER (Figure S2B), and 10 years for PR (Figure S2C). The ER+/HER2− subtype was associated with a better prognosis than the ER−/HER2+ subtype until 5 years after diagnosis when the curves crossed (Figure S2E). The ER−/HER2+ subtype and TNBC subtype survival curves plateaued after 5 years, but the survival curves of the ER+/HER2− subtype and ER+/HER2+ subtype kept declining after 5 years (Figure S2E).

### 3.3. Prognostic Factors for OS

Table 2 shows the univariate and multivariate Cox regression models with the primary outcome of OS. The risk was calculated as relative risk. The univariate analysis demonstrated that subtype, nuclear grade, locoregional therapy, and a number of organs with metastasis were significantly associated with OS. When other variables of interest were adjusted, the patients with ER+/HER2− subtype had a 71% higher risk of death than those with ER+/HER2+ subtype, and the patients with ER−/HER2+ subtype had a 49% higher risk of death than those with ER+/HER2+ subtype. Furthermore, the patients with TNBC had almost 4 times the risk of death in those with ER+/HER2+ disease. The patients with grade 3 disease had a 74% higher risk of death than those with grade 1 disease. The patients who received locoregional therapy had a 50% lower risk of death than those without locoregional therapy. The patients with metastasis to a single organ had a 36% lower risk of death than those with metastasis to multiple organs.

### 3.4. Changes in OS over Time

**Figure 2:**
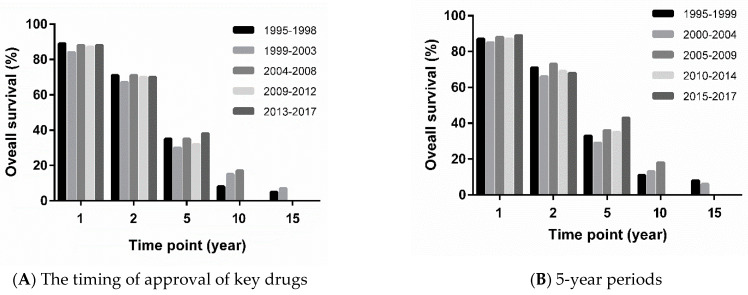
Overall survival rates for patients with de novo metastatic breast cancer at 1, 2, 5, 10, and 15 years after diagnosis according to diagnosis date, with dates grouped according to (**A**) the timing of approval of key drugs and (**B**) 5-year periods.

Figure 2 shows the OS rates for patients with dnMBC by diagnosis date, with dates grouped according to the timing of approval of key drugs (Figure 2A) and 5-year periods (Figure 2B). In both analyses, the 5-year and 10-year OS rates tended to improve over time, whereas the 1-year and 2-year OS rates did not. In the analysis based on the timing of approval of key drugs, the 5-year OS rate improved from 35% (95% CI: 24–46%) for patients diagnosed in 1995–1998 to 38% (95% CI: 32–44%) for those diagnosed in 2013–2017, and the 10-year OS rate improved from 8% (95% CI: 3–16%) for patients diagnosed in 1995–1998 to 17% (95% CI: 14–21%) for those diagnosed in 2004–2008. In the analysis based on 5-year periods, the 5-year OS rate improved from 33% (95% CI: 24–42%) for patients diagnosed in 1995–1999 to 43% (95% CI: 33–52%) for those diagnosed in 2015–2017, and the 10-year OS rate improved from 11% (95% CI: 6–18%) for patients diagnosed in 1995–1999 to 18% (95% CI: 14–21%) for those diagnosed in 2005–2009.

**Figure 3:**
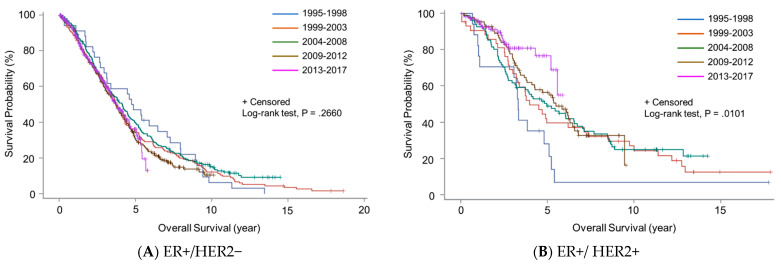

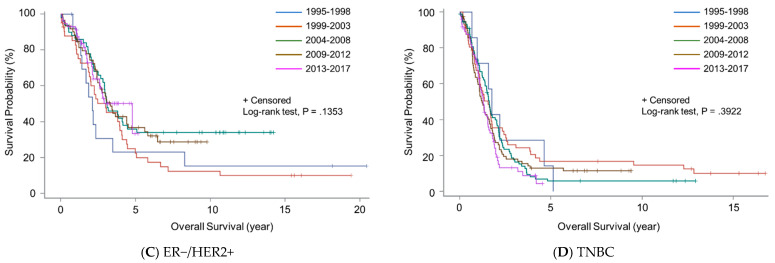
Overall survival curves for patients with de novo metastatic breast cancer according to year of diagnosis and breast cancer subtype: (**A**) estrogen receptor positive and HER2 negative (ER+/HER2−); (**B**) ER+/HER2 positive (HER2+); (**C**) ER-negative/HER2+; (**D**) triple-negative breast cancer.

Next, we investigated the change in OS over time stratified by breast cancer subtype. We did not observe any significant change in OS over time for ER+/HER2− subtype or TNBC (Figure 3A,D). In contrast, OS for the ER+/HER2+ subtype significantly improved over time (Figure 3B). Although the result was not statistically significant, OS for the ER−/HER2+ subtype tended to improve over time (Figure 3C).

## 4. Discussion

The present study showed that the 5-year and 10-year OS rates for patients with dnMBC tended to improve over time across time periods defined according to the dates of approval of key agents and across 5-year periods. In addition, OS improved significantly over time in the ER+/HER2+ subtype and tended to improve over time in the ER-/HER2+ subtype. Furthermore, we identified biological and clinical prognostic factors for dnMBC, including IBC status, ER status, PR status, HER2 status, subtype, nuclear grade, locoregional therapy, and a number of organs with metastasis.

Consistent with the results of a previous study of dnMBC versus rMBC [8], our study showed a tendency toward improvement in OS over time for dnMBC [8]; however, this was observed only 5 and 10 years after diagnosis. This result underscores the difficulty of improving the survival outcome for patients with dnMBC with progression within 2 years after diagnosis. In addition, our analysis revealed that many of the deaths within 2 years were in patients with TNBC, a subtype that did not show a significant improvement in OS over time.

TNBC does not have a specific druggable target. The PARP inhibitors could be a candidate for the treatment of TNBC; however, we estimated the contribution in terms of improving OS would be limited because the prevalence of *BRCA1/2* mutations in TNBC is approximately 10% to 20% in unselected cohorts [5,6,7]. A recent breakthrough in the treatment of metastatic TNBC is the use of an immune checkpoint inhibitor. The IMpassion130 trial showed that in patients with metastatic TNBC, PD-L1 blockade prolonged OS by approximately 4 months compared to OS in the control group [9]. Moreover, the subgroup of patients positive for PD-L1 by IHC demonstrated an improvement in OS of approximately 10 months with immune checkpoint inhibitor treatment. The survival benefit of immunochemotherapy with ICI is still under investigation in ongoing clinical trials, and the definitive results are expected to be available within the next 5 years. Given the current situation of drug development for metastatic TNBC, we expect that the novel treatment of dnMBC will contribute to the improvement of survival outcomes for TNBC in the next few years.

The subgroup analysis showed significant OS improvement for the ER+/HER2+ subtype and a tendency toward OS improvement for the ER−/HER2+ subtype over time. The OS improvement in HER2+ disease was due to HER2-targeted drugs, especially trastuzumab and pertuzumab. Those HER2-targeted drugs have revolutionized the treatment of HER2+ breast cancer and significantly prolonged survival. A systematic review of the efficacy of trastuzumab-containing regimens showed that they significantly decreased the HR for both death (HR, 0.82; 95% CI, 0.71–0.94; *p* = 0.004) and disease progression (HR, 0.61; 95% CI, 0.54–0.70; *p* < 0.00001) [4]. Moreover, the combination of trastuzumab and pertuzumab with docetaxel prolonged OS by approximately 16 months compared with the placebo combination group [10]. In contrast to the efficacy of HER2-targeted drugs against HER2+ breast cancer, the efficacy of fulvestrant against ER+ and/or PR+ MBC was limited to improvements in disease progression; fulvestrant as monotherapy improved median progression-free survival by approximately 3 months [11]. However, the combination of fulvestrant with CDK 4/6 inhibitor showed a significant improvement of OS, with HRs of 0.746 and 0.71, respectively, in the MONALEESA-2 and MONALEESA-7 trials [12,13]. Since the FDA approved CDK 4/6 inhibitor to treat metastatic HR+ breast cancer in 2015, we could not evaluate the survival effect of CDK 4/6 inhibitor in the present study. An updated analysis after the next 5 years will be needed to evaluate the survival effect of CDK 4/6 inhibitors in the real world.

As we expected, the clinical prognostic factors for dnMBC in the present study—subtype, nuclear grade, locoregional therapy, and number of organs with metastasis—were consistent with those previously reported [14,15]. Intriguingly, we observed that the survival curves for patients stratified by ER status and PR status crossed around 10 years. The survival curves of ER+ and PR+ groups kept declining and crossed the comparison group around 10 years after diagnosis. This result could be partly due to the acquired resistance to endocrine therapy during the long course of endocrine treatment. Once a tumor has acquired resistance, the selection of effective treatment becomes very difficult, and the survival outcome would be poor. In the present study, HR+ dnMBC had a better survival outcome than HER2+ dnMBC at baseline; however, overcoming resistance will be the key to further improve the survival outcome for the HR+ subgroup.

The multivariate analysis also showed that locoregional therapy and the number of organs with metastasis were prognostic factors. The efficacy of locoregional therapy for the primary tumor in patients with dnMBC has been discussed extensively. To date, three randomized clinical trials have been performed to answer the clinical question. A randomized controlled study from India with 716 patients with dnMBC found no survival benefit from early locoregional therapy [16]. In contrast, another randomized study by the Turkish Federation showed an improvement of OS by 17% with locoregional therapy [17]. The latest results of a large phase III randomized clinical trial with 256 patients with dnMBC (ECOG-ACRIN Research Group [E2108]) showed that locoregional therapy for the primary tumor did not significantly improve OS (3-year OS rate, 68.4% for the locoregional therapy group vs. 67.9% for the non-locoregional therapy group; *p* = 0.63 by log-rank test) [18]. The major cause for those controversial results would come from a selection bias. It would be highly probable that the patient with limited disease, including oligo-metastasis, dormant tumor, and high sensitivity to systemic therapy may benefit from locoregional therapy. Although we did not count the number or measure tumor size in each metastatic site, 62% of the patients in our dataset had a single-organ metastasis. The amount of tumor burden and the site of metastasis would impact the effect of locoregional therapy on the survival outcome in the present study. We expect to obtain further insights from the Japan Clinical Oncology Group (JCOG) ongoing clinical trial, which has a similar design to E2108 [19]. Overall, the impact of locoregional therapy on the primary tumor on survival appears to be limited at this moment; however, the benefit of locoregional therapy for controlling local disease progression should be investigated to improve the patient’s quality of life.

## 5. Limitations

The present study has several limitations. First, we excluded 756 of 2737 initially screened patients because of a lack of necessary pathological information. This process might have introduced a selection bias and affected the results. Second, we did not investigate the use of key drugs in each patient and did not evaluate the direct association between drug use and change in OS. Last, the methods of pathological evaluation, including the type of antibody, IHC procedure, and evaluation methods were not standardized throughout the study period. To overcome these limitations, a detailed evaluation via a well-designed prospective study would be crucial.

## 6. Conclusions

In this large retrospective cohort study, we confirmed that dnMBC tended to improve in OS over time, especially at the time points of 5 and 10 years after diagnosis. Although the improvement was not large, the significant OS improvement in the ER+/HER2+ subtype and the tendency toward OS improvement in the ER-/HER2+ subtype suggested the contribution of HER2-targeted therapy. The biological and clinical prognostic factors for dnMBC were similar to those described in previous reports, however, further investigation will be needed to elucidate the biology of dnMBC.

## Figures and Tables

**Table 1:** Patient characteristics (N = 1981).

Characteristic		Number of Patients (%)
Year of diagnosis, grouping 1 (based on the date of approval of key agents)	1995–1998	72 (4)
	1999–2003	305 (15)
	2004–2008	469 (24)
	2009–2012	564 (29)
	2013–2017	571 (29)
Year of diagnosis, grouping 2	1995–1999	100 (5)
(5-year periods)	2000–2004	353 (18)
	2005–2009	517 (26)
	2010–2014	676 (34)
	2015–2017	335 (17)
Age at diagnosis, median (range), year		52 (22–93)
ER status	Positive	1398 (71)
	Negative	580 (29)
	Unknown	3
PR status	Positive	1051 (53)
	Negative	920 (47)
	Unknown	10
HER2 status	Positive	555 (28)
	Negative	1426 (72)
Subtype	ER+/HER2+	330 (17)
	ER+/HER2−	1120 (57)
	ER−/HER2+	225 (11)
	TNBC	306 (15)
Nuclear grade	1	96 (5)
	2	754 (38)
	3	1131 (57)
IBC	Yes	339 (17)
	No	1642 (83)
Locoregional therapy	Yes	775 (39)
	No	1206 (61)
Metastasis to a single organ	Yes	1227 (62)
	No	754 (38)

ER, estrogen receptor; IBC, inflammatory breast cancer; PR, progesterone receptor; TNBC, triple-negative breast cancer.

**Table 2:** Results of univariate and multivariate analysis for overall survival.

Variables		HR (95% CI)	*p*-Value	HR (95% CI)	*p*-Value
Year of diagnosis, grouping 1	1995–1998 vs. 2013–2017	1.12 (0.85–1.48)	0.4117		
	1999–2003 vs. 2013–2017	1.11 (0.92–1.32)	0.2683		
	2004–2008 vs. 2013–2017	1 (0.85–1.18)	0.9985		
	2009–2012 vs. 2013–2017	1.07 0(.91–1.26)	0.3952		
Year of diagnosis, grouping 2	1995–1999 vs. 2000–2004	0.91 (0.72–1.15)	0.4188		
	2005–2009 vs. 2000–2004	0.8 (0.69–0.93)	0.0029		
	2010–2014 vs. 2000–2004	0.91 (0.78–1.05)	0.1857		
	2015–2017 vs. 2000–2004	0.88 (0.7–1.1)	0.2541		
Age at diagnosis		1.008 (1.004–1.013)	0.0002		
ER status	Positive vs. Negative	0.59 (0.53–0.59)	<0.0001		
PR status	Positive vs. Negative	0.64 (0.57-0.64)	<0.0001		
HER2 status	Positive vs. Negative	0.64 (0.56–0.64)	<0.0001		
Subtype	ER+/HER2− vs. ER+/HER2+	1.54 (1.31–1.54)	<0.0001	1.71 (1.45–2.02)	<0.0001
	ER−/HER2+ vs. ER+/HER2+	1.41 (1.13–1.41)	<0.0001	1.49 (1.2–1.87)	0.0004
	TNBC vs. ER+/HER2+	3.7 (3.05–3.7)	<0.0001	3.93 (3.23–4.78)	<0.0001
Nuclear grade	2 vs. 1	1.07 (0.82–1.07)	0.0023	1.21 (0.92–1.6)	0.1677
	3 vs. 1	1.51 (1.16–1.51)	<0.0001	1.74 (1.32–2.29)	<0.0001
IBC	Yes vs. No	1.18 (1.03–1.18)	0.0212		
Locoregional therapy	Yes vs. No	0.53 (0.47–0.53)	<0.0001	0.5 (0.45–0.56)	<0.0001
Metastasis to a single organ	Yes vs. No	0.59 (0.53–0.59)	<0.0001	0.64 (0.58–0.72)	<0.0001

ER, estrogen receptor; IBC, inflammatory breast cancer; PR, progesterone receptor; TNBC, triple-negative breast cancer.

## Data Availability

The data of the present study is not publicly available.

## Supplementary Materials

The following are available online at https://www.mdpi.com/article/10.3390/cancers13112650/s1, Figure S1. Kaplan-Meier curve of overall survival for all dnMBC patients. Figure S2. Kaplan-Meier curve of overall survival stratified by IBC (A), ER (B), PR (C), HER2 (D), subtypes (E), grade (F), the locoregional therapy (G) and single or multiple organ metastasis (H).
