# Supplementary material for: Changes in Overall Survival over Time for Patients with de novo Metastatic Breast Cancer
Source: Cancers (Basel). 2021 May 28;13(11):2650. doi: 10.3390/cancers13112650 (PMC8198851; doi:10.3390/cancers13112650)
Supplement: Supplementary file 1 [file cancers-13-02650-s001.zip › cancers-1226386-supplementary.pdf]

SUPPLEMENTARY FIGURES

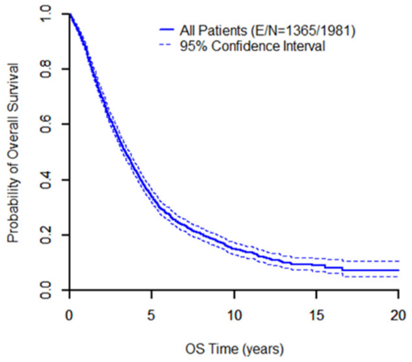

Figure S1. Kaplan-Meier curve of overall survival for all dnMBC patients

Figure S2.

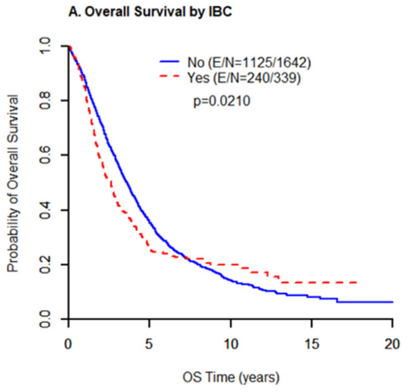

A. Kaplan-Meier curve of overall survival stratified by IBC

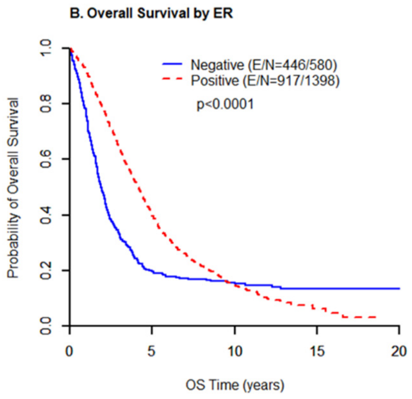

B. Kaplan-Meier curve of overall survival stratified by ER

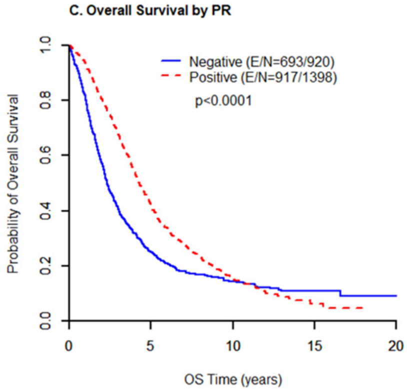

C. Kaplan-Meier curve of overall survival stratified by PR

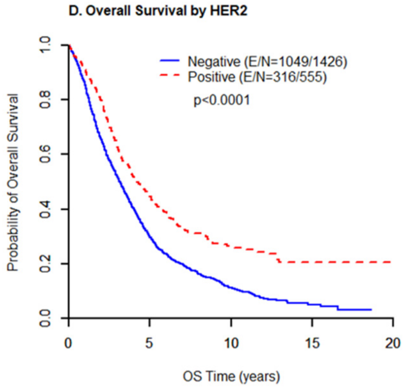

D. Kaplan-Meier curve of overall survival stratified by HER2

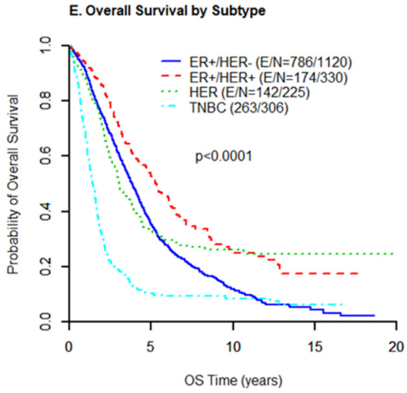

E. Kaplan-Meier curve of overall survival stratified by subtypes

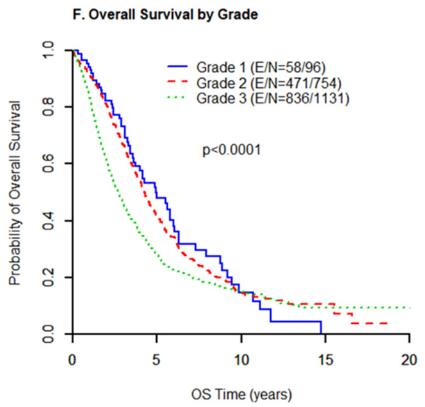

F. Kaplan-Meier curve of overall survival stratified by grade

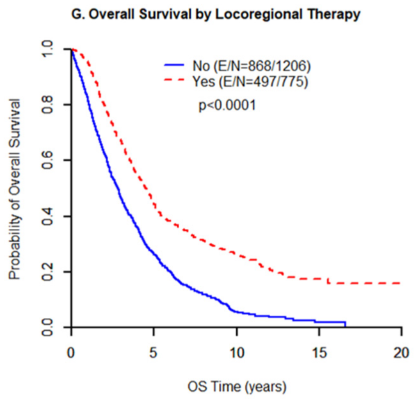

G. Kaplan-Meier curve of overall survival stratified by the locoregional therapy

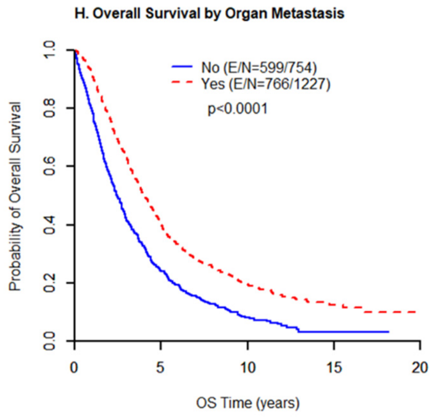

H. Kaplan-Meier curve of overall survival stratified by single or multiple organ metastasis
